# Trajectory Tracking with Obstacle Avoidance for Nonholonomic Mobile Robots with Diamond-Shaped Velocity Constraints and Output Performance Specifications

**DOI:** 10.3390/s24144636

**Published:** 2024-07-17

**Authors:** Panagiotis S. Trakas, Spyridon I. Anogiatis, Charalampos P. Bechlioulis

**Affiliations:** Division of Signals and Control Systems, Department of Electrical and Computer Engineering, University of Patras, Rio, 26504 Patras, Greece; ptrakas@upatras.gr (P.S.T.); anogiatiss@ac.upatras.gr (S.I.A.)

**Keywords:** adaptive performance control, input constraints, target tracking, collision avoidance

## Abstract

In this paper, we address the trajectory-/target-tracking and obstacle-avoidance problem for nonholonomic mobile robots subjected to diamond-shaped velocity constraints and predefined output performance specifications. The proposed scheme leverages the adaptive performance control to dynamically adjust the user-defined output performance specifications, ensuring compliance with input and safety constraints. A key feature of this approach is the integration of multiple constraints into a single adaptive performance function, governed by a simple adaptive law. Additionally, we introduce a robust velocity estimator with a priori-determined performance attributes to reconstruct the unmeasured trajectory/target velocity. Finally, we validate the effectiveness and robustness of the proposed control scheme, through extensive simulations and a real-world experiment.

## 1. Introduction

The field of mobile robotics has experienced significant advancements in recent years, driven by the increasing demand for autonomous systems capable of performing complex tasks in dynamic environments. A critical aspect of mobile robotics is the ability to accurately track trajectories and avoid obstacles, particularly for nonholonomic mobile robots. These robots are subject to motion constraints, typically due to their wheeled design, which restricts their instantaneous movement in every direction, thereby adding complexity to their control and navigation.

Trajectory tracking involves guiding a mobile robot along a specified trajectory, often represented by a reference signal. This reference signal can be generated from various sources, such as predefined paths, waypoints, or dynamically changing objectives, e.g., a moving target. The robot’s primary objective is to closely follow this reference signal while adapting its motion to external factors such as obstacles, changes in terrain, or unexpected disturbances. Achieving accurate trajectory tracking is essential for ensuring that the robot effectively accomplishes its tasks. Whether navigating through a cluttered environment, following a curved path, or maintaining a specific speed profile, the robot must continuously adjust its motion to stay on course. However, the real world is rarely static or predictable. Obstacles, both static and dynamic, pose significant challenges to trajectory tracking. Static obstacles, such as walls, furniture, or terrain features, require the robot to plan its path around them to avoid collisions. Dynamic obstacles, like moving vehicles or pedestrians, demand even greater agility and responsiveness from the robot to navigate safely while adhering to its trajectory. To address these challenges, robust control strategies are essential. These strategies may utilize sensors, such as LiDARs, cameras, or sonars, to detect obstacles in the robot’s vicinity. Once detected, the robot must analyze this information and make real-time decisions to adjust its trajectory accordingly, either by deviating from the original path or by slowing down to allow the path to get clear.

It should be noted that the integration of trajectory tracking and obstacle avoidance is critical for the operation of mobile robots in various real-world scenarios. In urban environments, robots must navigate through crowded streets while avoiding pedestrians and vehicles. In search and rescue missions, they must traverse rough terrain and debris while evading obstacles to reach victims efficiently. In industrial automation settings, robots need to maneuver around machinery and obstacles while maintaining precise trajectories to perform tasks accurately and safely.

### 1.1. Related Literature

A broad range of studies have proposed innovative solutions for trajectory tracking and obstacle avoidance in various robotic systems, over the past years. For instance, the authors in [1] focus on target tracking guidance for unmanned surface vehicles, integrating obstacle avoidance capabilities through a bias proportional navigation guidance law. Similarly, Ref. [2] introduces an adaptive control approach for trajectory tracking and obstacle avoidance in mobile robots, employing a sliding mode observer and a stable tracking control law. The work in [3] proposes a fuzzy controller for nonholonomic mobile robot trajectory tracking and obstacle avoidance, which was proven to be efficient in dynamic environments. Furthermore, Ref. [4] studies the design of an obstacle avoidance controller based on nonlinear model predictive control for autonomous vehicle navigation, ensuring real-time trajectory tracking and collision avoidance, with the use of a sigmoid function for the reference trajectory and a risk index for collision avoidance. A multi-switching tracking-control scheme for autonomous mobile robots in unknown obstacle environments, combining trajectory tracking and obstacle avoidance controllers, was proposed in [5]. For nonholonomic two-wheeled mobile robots, [6] presents a method that uses either a proportional–integral (PI) controller or a fuzzy logic controller (FLC) for trajectory tracking, determined by a high-level planner. This method also employs a fuzzy controller to adjust the tracking controller’s actions in response to moving obstacles, addressing uncertainties effectively.

Further contributions include a robust nonlinear model predictive control (NMPC) scheme that was presented in [7] for underactuated Autonomous Underwater Vehicles (AUVs). The control approach ensures that the AUV follows a desired 3D trajectory while avoiding obstacles, despite uncertainties such as ocean currents and waves. The control system includes an online component solving a finite-horizon optimal control problem for nominal dynamics and an offline-tuned state feedback law ensuring that real trajectories remain within a predefined hyper-tube around the nominal path. In the same direction, Ref. [8] proposes a nonlinear model predictive controller for mobile robots, which calculates an optimal control sequence in real time to minimize tracking errors over a specified horizon. The controller adjusts the robot’s path in response to obstacles detected by range sensors, balancing between obstacle avoidance and tracking accuracy. The performance of the controller is influenced by the optimization horizon and the cost weights assigned to different types of tracking errors. Moreover, Ref. [9] introduces a nonlinear control scheme for tracking a moving target with nonholonomic ground vehicles, focusing on maintaining specific distance and bearing angle constraints. The controller utilizes a Barrier Lyapunov Function (BLF) to ensure that the tracking error converges near zero in finite time while adhering to the constraints. The authors in [10] propose a nonlinear control scheme for a wheeled mobile robot with nonholonomic constraints, aimed at achieving precise tracking and effective obstacle avoidance. The control strategy includes an extended state observer for estimating disturbances and velocity and a nonlinear controller that ensures the convergence of tracking errors and obstacle avoidance.

Regarding the constrained control framework, Ref. [11] introduces a trajectory-planning approach that incorporates multiple constraints such as robot motion speed and motion state, alongside dynamic obstacles. The method utilizes a time elastic band and a workspace potential field to establish the optimal robot speed and a costmap for detecting dynamic obstacles. The approach aims to achieve collision-free, smooth motion in mobile robots, demonstrating satisfactory obstacle avoidance and improved kinematics characteristics through the experimental results. The paper [12] proposes a parametric trajectory-planning scheme for mobile robots to navigate efficiently in environments with moving obstacles. The method involves formulating collision avoidance conditions as constraints and solving an unconstrained optimization problem to derive a feasible collision-free trajectory. The control torques necessary for the robot’s movement are calculated based on the dynamic model and the derived trajectory. Alternatively, ref. [13] presents a method based on nonlinear model prediction for trajectory tracking and obstacle avoidance for nonholonomic mobile robots. This method integrates collision avoidance as a nonlinear constraint in the trajectory-tracking-control problem, adapting to dynamic environments. In the work [14], an adaptive performance-control scheme is introduced for small fixed-wing UAVs to achieve longitudinal motion tracking, taking into account both input and state constraints.

Recently, the authors in [15] proposed a cost-effective observer-based control scheme for nonholonomic wheeled mobile robots (WMRs). This scheme relies solely on position and velocity measurements, eliminating the need for direct attitude measurements and ensuring robust trajectory tracking even in harsh environments. Complementarily, Ref. [16] develops a model predictive control (MPC) strategy that incorporates an adaptive artificial potential field (APF) for autonomous vehicle collision avoidance, facilitating smooth and safe navigation while reducing computational burden. Additionally, Ref. [17] introduces a high-speed vision system for non-contact measurement of wheel behavior, enabling precise analysis of slip and deformation during vehicle movement. This attribute is crucial for enhancing the tire design and vehicle dynamics. Furthermore, a study on the design and control of a digital twin system for a WMR is presented in [18]. This system consists of four main components: the physical WMR, the virtual WMR, data processing, and the application service. The paper [19] presents a single-stage adaptive controller for Euler–Lagrange systems with nonholonomic constraints, offering a simpler design compared to traditional double-stage controllers. This approach achieves stability in both the original and internal states without requiring direct access to the internal states, a common necessity in existing single-stage mechanisms.

In [20], an event-triggered model predictive control (EMPC) strategy is developed for WMRs to achieve effective trajectory tracking and obstacle avoidance. This strategy incorporates a potential field within the cost function to ensure smooth navigation and utilizes an adaptive prediction horizon to reduce computational demands. Additionally, an event-triggered mechanism is designed to decrease the frequency of solving the optimal control problem, thereby enhancing computational efficiency. The study in [21] presents a secure adaptive trajectory-tracking-control scheme for uncertain nonlinear robot systems operating in environments with multiple dynamic obstacles. This scheme ensures both obstacle avoidance and tracking performance, even within obstacle sensing regions and in the presence of unknown nonlinear uncertainties. This is achieved through the use of an integral–multiplicative Lyapunov barrier function and an adaptive mechanism to compensate for uncertainties. Moreover, Ref. [22] introduces an adaptive finite-time tracking-control scheme for autonomous vehicles, addressing challenges such as dynamic couplings, disturbances, and actuator saturation. The paper proposes a fuzzy logic system to manage lumped disturbances and an adaptive algorithm that adjusts gain online without prior knowledge of disturbance bounds. An auxiliary system is also developed to handle actuator saturation. This method ensures finite-time error convergence, however without compromising performance guarantees. In [23], a study is presented on path following for WMRs, utilizing an online optimization-based guidance vector field to address nonholonomic constraints and unknown disturbances. Similarly, Ref. [24] introduces a reinforcement learning-based adaptive control algorithm designed to tackle the tracking problem in WMRs with state and input time delays. This study transforms the typical WMR model into an affine nonlinear discrete-time system, incorporating a delay matrix function and Lyapunov–Krasovskii functionals to effectively manage delays. The adaptive control strategy employs radial basis function neural networks to ensure the uniform ultimate boundedness of all system signals and the convergence of tracking errors to a small compact set near zero. Additionally, Ref. [25] presents a novel collision-free tracking framework for quadruped robots, enhancing dynamic target tracking in obstacle-cluttered environments. This framework integrates a modified guidance vector field with a disturbance rejection controller, featuring a variable property vector that transforms elliptic integral curves into straight lines aimed at the target, thereby improving directness. Furthermore, the work in [26] addresses the challenge of motion planning and control in environments cluttered with obstacles, focusing on the integration of temporal and input constraints. This study introduces a novel hybrid control algorithm that learns to execute high-level objectives expressed as timed temporal logic formulas, combined with a motion controller for secure navigation within minimal time intervals. In [27], a nonlinear switched model predictive control (MPC) strategy is explored for trajectory tracking and obstacle avoidance in nonholonomic systems. This strategy incorporates a potential field in the cost function to ensure smooth path navigation and employs a switched mechanism with multiple Lyapunov functions to enhance switching stability. Additionally, the paper introduces an average dwell time to balance safety and stability throughout the control process. Similarly, Ref. [28] presents an approach for obstacle avoidance and trajectory tracking in autonomous electric vehicles using EMPC with an adaptive APF. The authors introduce an adaptive APF cost function to ensure obstacle avoidance and stability, alongside an event-triggered mechanism to reduce computational burden. Finally, Ref. [29] develops a path-following control algorithm for nonholonomic mobile robots, utilizing a guiding vector field to design a nonlinear motion controller. The vector field is based on a predefined smooth function and the robot’s kinematic model, with its integral curves converging to the desired trajectory.

Despite the significant advancements highlighted earlier, there remains an ongoing challenge in the literature regarding the holistic integration of multiple constraints for mobile robots. It is essential to achieve this integration while maintaining low computational complexity and high robustness to enhance applicability in real-world scenarios. Current approaches often address individual constraints or trade-offs between performance and robustness, leaving opportunities for innovation in developing unified solutions that concurrently impose multiple constraints such as input–output constraints, safety considerations, and feedback limitations. This challenge emphasizes the need for further research to bridge this gap and advance the development of versatile control strategies for nonholonomic robots.

### 1.2. Contributions

In this work, we consider the trajectory-tracking-control problem with obstacle avoidance for kinematic robotic agents subject to nonholonomic constraints, output performance characteristics, and limited control capacity. The objective of the agent is to track a reference trajectory or a target, while avoiding any collisions with either static or moving obstacles. User-defined performance specifications are adapted online, based on the adaptive performance control (APC) methodology presented in [30], to meet the input and safety constraints. The main contributions of this paper are outlined as follows:

- Contrary to the related literature, we impose trajectory tracking with adaptive performance specifications, incorporating multiple constraints, including obstacle avoidance and diamond-shaped input limitations. Notably, the proposed control scheme is distinguished by its low complexity and straightforward gain selection.
- Given the inherent conflicts among multiple constraints, we introduce a novel adaptation mechanism that governs the performance specifications, thereby ensuring the robot’s safe navigation. In this way, multiple operational and safety constraints are incorporated into a single adaptive performance function.
- We introduce a novel robust velocity estimator with predefined performance attributes to reconstruct the velocity of the reference trajectory/target.

## 2. Problem Formulation

**Figure 1:**
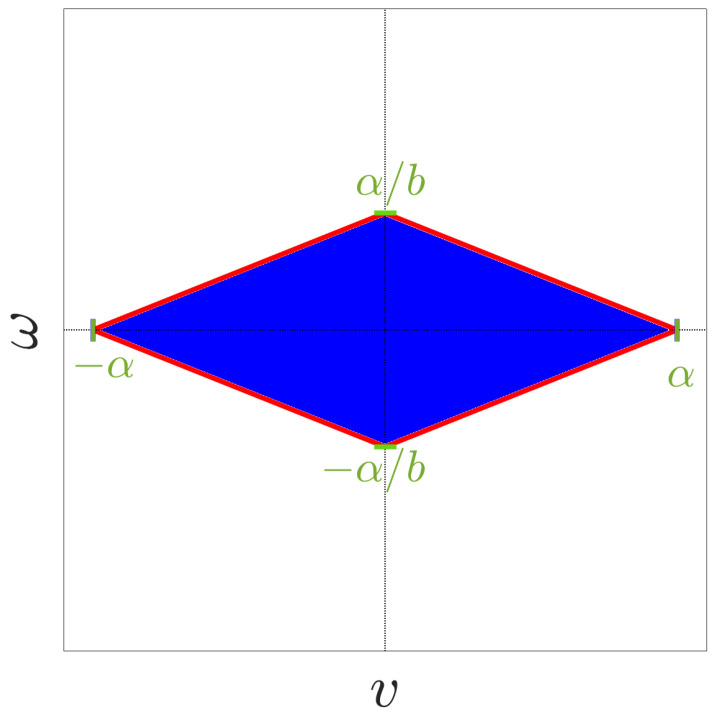
Diamond-shaped constraints for the unicycle model (1). The blue area denotes the compact set U.

In this work, we consider a disk-shaped robot with radius *R*, operating within a 2D space that includes both static and moving obstacles. The position of the robot center is denoted by $p={\left[x,y\right]}^{T}\in R^{2}$, and its motion is governed by the following nonholonomic kinematic model:

(1) $$\begin{array}{cc} \dot{x} & =v\cos\theta ,\\ \dot{y} & =v\sin\theta ,\\ \dot{\theta } & =\omega , \end{array}$$

where *v* and ω represent the linear and angular velocities, respectively. These velocities are the control inputs of the system, denoted compactly as $u={\left[v,\omega \right]}^{T}\in U$. Due to physical limitations, the robot’s combined motion is constrained within the following compact set:

(2) U{(v,ω):|v/α|+|bω/α|≤1},

where α is the maximum wheel velocity and *b* represents half the distance between the two driving wheels. The diamond-shaped constraints are visualized in Figure 1.

Moreover, let $p_{d}\left(t\right)={\left[x_{d},y_{d}\right]}^{T}\in R^{2}$ denote either a smooth reference trajectory generated by an exosystem or the position of a moving target within the 2D space.

The objective of this work is to design a control protocol for the input-constrained nonholonomic robot described by (1), ensuring that it tracks the reference trajectory $p_{d}\left(t\right)$ with adaptive performance specifications for all time. By adaptive performance specifications, we mean that the convergence rate and the maximum steady-state value of the tracking error are predetermined by the user and are dynamically adjusted online to meet hard constraints, including input saturation and collision avoidance. To solve the trajectory-tracking problem, the robot is equipped with appropriate onboard sensors (e.g., LiDAR, proximity sensors) to detect the obstacles and uses a dedicated target-tracking system (e.g., vision) to obtain the reference position $p_{d}\left(t\right)$, which is assumed continuously available for measurement.

## 3. Robust Velocity Estimator

In this section, we introduce a novel velocity estimator with prescribed transient and steady-state attributes that regulate the estimation performance. First, we define the mapping $T\frac{1}{2}\ln\left(\frac{1+\chi }{1-\chi }\right)$ and its derivative as $J\frac{1}{1-\chi^{2}}$. Note that T:(−1,1)→(−∞,∞) is a strictly increasing and radially unbounded function of its argument. The proposed estimator, which reconstructs the derivative of a continuous signal χ(t), is given by:

(3) $$\begin{array}{cc} {\dot{z}}_{1} & =a_{1}J\left(\frac{\chi -z_{1}}{\rho_{o_{1}}}\right)T\left(\frac{\chi -z_{1}}{\rho_{o_{1}}}\right),\;a_{1}>0 \end{array}$$

(4) $$\begin{array}{cc} {\dot{z}}_{2} & =a_{2}J\left(\frac{{\dot{z}}_{1}-z_{2}}{\rho_{o_{2}}}\right)T\left(\frac{{\dot{z}}_{1}-z_{2}}{\rho_{o_{2}}}\right),\;a_{2}>0 \end{array}$$

where:

$$\begin{array}{c} \rho_{o_{i}}\left(t\right)=\left(\rho_{o_{i}}\left(0\right)-\rho_{o}^{\infty }\right)\exp\left(-\lambda_{o}t\right)+\rho_{o}^{\infty },\;i=1,2, \end{array}$$

with $\rho_{o_{1}}\left(0\right)>\left|\chi \left(0\right)-z_{1}\left(0\right)\right|$, $\rho_{o_{2}}\left(0\right)>\left|{\dot{z}}_{1}\left(0\right)-z_{2}\left(0\right)\right|$, and $\lambda_{o},\rho_{o}^{\infty }$ positive constants encapsulating the desired transient and steady-state performance specifications of the proposed estimator, respectively. Notice that $z_{1}\left(t\right)$ denotes the estimate of the measured signal χ(t) and $z_{2}\left(t\right)$ denotes the estimate of its unmeasured derivative $\dot{\chi }$. This formulation ensures that the estimation performance in terms of both transient and steady-state behavior can be implicitly determined by the selection of the aforementioned parameters.

**Corollary** **1.** *Consider a bounded and continuous reference signal $\chi :R_{+}\to R$ with an unknown, but bounded first derivative. The tracking differentiator (3) and (4) is exponentially $\rho_{o}^{\infty }$-convergent with rate $\lambda_{o}$ in the sense that the estimation errors $\chi \left(t\right)-z_{1}\left(t\right)$ and $\dot{\chi }\left(t\right)-z_{2}\left(t\right)$ converge to regions around the origin faster than $\exp(-\lambda_{o}t)$, with absolute steady-state error of order $O(\rho_{o}^{\infty })$.*

**Proof.**  The proof proceeds by establishing the existence and uniqueness of a maximal solution ${\left[\xi_{1}\left(t\right),\xi_{2}\left(t\right),z_{1}\left(t\right),z_{2}\left(t\right)\right]}^{T}$ over the interval $\left[0,t_{\max}\right)$, where $\xi_{1}\left(t\right)\frac{\chi \left(t\right)-z_{1}\left(t\right)}{\rho_{o_{1}}\left(t\right)}$ and $\xi_{2}\left(t\right)\frac{{\dot{z}}_{1}\left(t\right)-z_{2}\left(t\right)}{\rho_{o_{2}}\left(t\right)}$. Initially, $\rho_{o_{1}}\left(0\right)$ and $\rho_{o_{2}}\left(0\right)$ are chosen such that $\xi_{1}\left(0\right)\in \left(-1,1\right)$ and $\xi_{2}\left(0\right)\in \left(-1,1\right)$, ensuring the boundedness of the loop signals. Since the differentiator dynamics are continuous and locally Lipschitz in $\left(\xi_{1},\xi_{2},z_{1},z_{2}\right)$, we can easily establish the existence of a unique solution over the domain $\left(-1,1\right)\times \left(-1,1\right)\times R^{2}$. By extending arguments from Theorem 2 in [31], we establish that $\xi_{1}\left(t\right)$ and $\xi_{2}\left(t\right)$ remain within (−1,1) for all t≥0. The aforementioned result implies that the solution $z_{1}\left(t\right)$ and $z_{2}\left(t\right)$ of the tracking differentiator (3) and (4) remains bounded for all t≥0. This ensures that $|\chi \left(t\right)-z_{1}\left(t\right)|<\rho_{o_{1}}\left(t\right)$ and $|{\dot{z}}_{1}\left(t\right)-z_{2}\left(t\right)|<\rho_{o_{2}}\left(t\right)$. In conclusion, the proof establishes that the tracking differentiator (3) and (4) exhibits exponential $\rho_{o}^{\infty }$-convergence with rate $\lambda_{o}$. This means that the estimation error $\lim_{t\to \infty }\left|\dot{\chi }\left(t\right)-z_{2}\left(t\right)\right|$ is $O(\rho_{o}^{\infty })$ with the convergence rate determined by $\exp(-\lambda_{o}t)$.□

**Remark** **1.** *The velocity estimator can be employed to accurately estimate ${\dot{p}}_{d}\left(t\right)$ based on the available measurements, i.e., $p_{d}\left(t\right)$, ensuring that the robot can effectively track the reference trajectory despite the absence of direct velocity measurements. Since $p_{d}\left(t\right)={\left[x_{d}\left(t\right),y_{d}\left(t\right)\right]}^{T}\in R^{2}$, we have to leverage the velocity estimator twice to independently reconstruct ${\dot{x}}_{d}\left(t\right)$ and ${\dot{y}}_{d}\left(t\right)$.*

## 4. Control Architecture

In this section, we present the design procedure of the proposed controller that effectively addresses the problem stated in Section 2. Due to the nonholonomic constraints, the center point of the robot cannot move arbitrarily. Therefore, we first employ a linearization method to transform the unicycle model into a single integrator model. Subsequently, we design the tracking controller to impose input, output, and safety constraints.

### 4.1. Feedback Linearization of Nonholonomic Model

**Figure 2:**
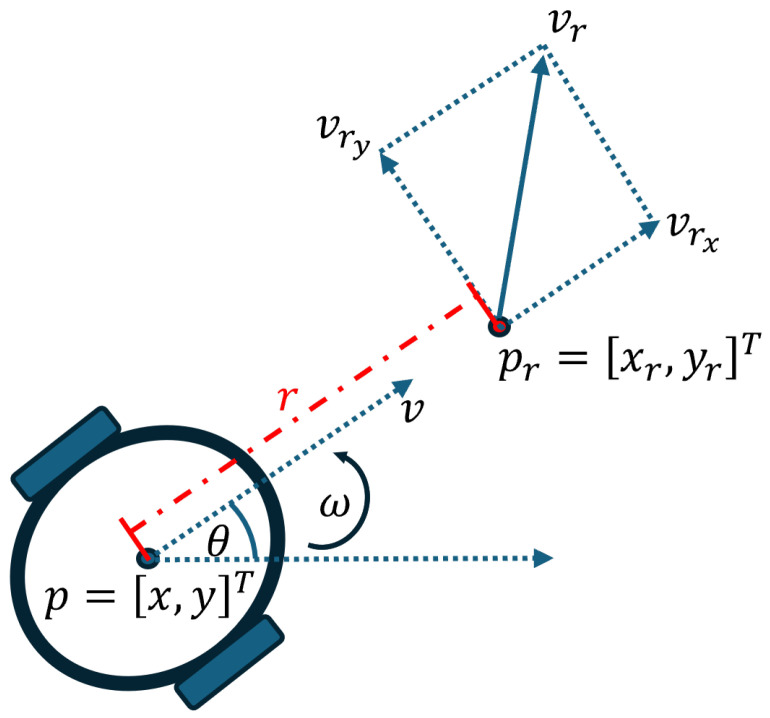
Feedback linearization of the non-affine model (1).

The model (1) is nonlinear. Inspired by [32], instead of considering the center point of the unicycle robot, we focus on a point shifted from the center by *r*, as illustrated in Figure 2 and described by:

(5) $$\begin{array}{cc} x_{r} & =x+r\cos\theta , \end{array}$$

(6) $$\begin{array}{cc} y_{r} & =y+r\sin\theta . \end{array}$$

Thus, the motion of the shifted point is governed by the following holonomic model:

(7) $$\begin{array}{c} {\dot{p}}_{r}=u_{r}, \end{array}$$

where $u_{r}\in R^{2}$ denotes the new input to be designed. Note that $u_{r}$ is simply the velocity of the offset point. Consequently, the model of the offset point becomes a linear single-integrator model. Once $u_{r}$ is designed, the original inputs *v* and ω can be calculated as:

(8) $$\begin{array}{c} \left[\begin{array}{c} v\\ \omega \end{array}\right]=G^{-1}\left(\theta \right)u_{r}, \end{array}$$

with $G\left(\theta \right)=\left[\begin{array}{cc} \cos\theta & -r\sin\theta \\ \sin\theta & r\cos\theta \end{array}\right]$ which is invertible for r>0.

**Remark** **2.** *While the center point of the robot cannot slide sideways, the offset point behaves like a single-integrator system. Leveraging this feedback linearization approach, we aim at controlling the position of the offset point instead of the center point. For a small r, this methodology is viable in practice as p and $p_{r}$ are closely aligned. It is crucial to note that $u_{r}$ typically relies on specific measurements. To ensure accurate control of the offset point, we have to measure the relative position with respect to the offset point, rather than relying on measurements from the center point. This approach ensures that the offset point converges to the desired position, effectively aligning the center point with the target position.*

### 4.2. Control Signal Design

*Step 1.* Let us denote the performance function ρ(t), which encapsulates the transient and steady-state performance specifications for the tracking error $e\left(t\right)=∥p_{d}\left(t\right)-p_{r}\left(t\right)∥$. Additionally, let us define the set of nearby obstacles as O, which are detected by the onboard sensors. We assume that a nearby obstacle is detected if it is located within a distance $\delta_{r}=\delta +R+r_{o}$ from the robot, where $r_{o}$ denotes the radius of the circular obstacle and δ is a small positive constant. Specifically, j∈O if $∥p_{r}-p_{o_{j}}∥\leq \delta_{r}$, where $p_{o_{j}}\in R^{2}$ denotes the center of obstacle *j*.

*Step 2.* Next, we design the reference velocity $u_{r}$ for the offset point $p_{r}$ as follows:

(9) $$\begin{array}{c} u_{r}=k_{d}T\left(\frac{e}{\rho }\right)e+{\hat{v}}_{d}+\lambda \left(\rho -\rho_{\infty }\right)\frac{p_{d}-p_{r}}{\rho }+k_{o}u_{c}, \end{array}$$

where $e=\frac{p_{d}-p_{r}}{∥p_{d}-p_{r}∥}$ is the unit vector of the tracking error *e*, $T\frac{1}{2}\ln\left(\frac{1+\frac{e}{\rho }}{1-\frac{e}{\rho }}\right)$, and ${\hat{v}}_{d}={\left[z_{2}^{x},z_{2}^{y}\right]}^{T}$ denotes the estimate of the reference velocity ${\dot{p}}_{d}={\left[{\dot{x}}_{d},{\dot{y}}_{d}\right]}^{T}$, obtained using the proposed velocity estimator (3) and (4) with inputs $x_{d}$ and $y_{d}$, respectively. Additionally, $u_{c}$ is a collision avoidance term [33] given by:

(10) $$\begin{array}{c} u_{c}=\sum_{j\in O}\left[\left(\frac{1}{∥p_{r}-p_{o_{j}}∥-\left(R+r_{o}\right)}-\frac{1}{\delta_{r}-\left(R+r_{o}\right)}\right)\left(p_{r}-p_{o_{j}}\right)\right], \end{array}$$

representing the repulsive velocity that keeps the robot away from nearby obstacles, thereby preventing imminent collisions. Invoking (8), the reference velocity for the center of the robot can be obtained by:

(11) $$\begin{array}{c} u_{d}=G^{-1}\left(\theta \right)u_{r} \end{array}$$

*Step 3.* In step 2, we designed the reference control signal $u_{d}={\left[v_{d}\left(t\right),\omega_{d}\left(t\right)\right]}^{T}\in R^{2}$ to ensure safety and satisfy the output constraints. However, since $u_{d}\left(t\right)$ must lie within the compact set U, we introduce a saturation function to generate a feasible control input that adheres to the diamond-shaped hard input constraints. By selecting $\bar{v}=\alpha$ and $\bar{\omega }=\alpha /b$ as the saturation levels for the linear and angular velocities, respectively, we adopted a saturation function σ(·):(−∞,∞)×(−∞,∞)→U. This function maps the desired control signal $u_{d}\notin U$ to the boundary of the set $U=\left[-\bar{v},\bar{v}\right]\times \left[-\bar{\omega },\bar{\omega }\right]$ based on the radial distance of $u_{d}$ from the origin. Hence, the actual control input is given as:

(12) $$\begin{array}{c} u\sigma \left(v,\omega \right)=\left\{\begin{array}{cc} \left(v,\omega \right) & \mathrm{if}\;(w_{1},w_{2},w_{3})\in W\\ \left(\frac{v}{w_{2}+w_{3}},\frac{\omega }{w_{2}+w_{3}}\right) & \mathrm{if}\;v\neq 0\wedge \omega \neq 0\wedge (w_{1},w_{2},w_{3})\notin W\\ \left(v^{m},\omega^{m}\right) & \mathrm{otherwise} \end{array}\right. \end{array}$$

where:

$$\begin{array}{cc} \; & w_{1}=\frac{-v\cdot \bar{\omega }\cdot \mathrm{sgn}\left(\omega \right)-\omega \cdot \bar{v}\cdot \mathrm{sgn}\left(v\right)+\alpha }{\alpha }\\ \; & w_{2}=\frac{v\cdot \bar{\omega }\cdot \mathrm{sgn}\left(\omega \right)}{\alpha },\;\;w_{3}=\frac{\omega \cdot \bar{v}\cdot \mathrm{sgn}\left(v\right)}{\alpha }\\ \; & \alpha =\bar{v}\cdot \bar{\omega }\cdot \mathrm{sgn}\left(v\right)\cdot \mathrm{sgn}\left(\omega \right)\\ \; & u^{m}=\min(|v|,\bar{v})\cdot \mathrm{sgn}\left(v\right)\\ \; & \omega^{m}=\min(|\omega |,\bar{\omega })\cdot \mathrm{sgn}\left(\omega \right) \end{array}$$

and the open set W(0,1)×(0,1)×(0,1).

Finally, we incorporated input–output and safety constraints in a single adaptive law that dictates the evolution of the performance function:

(13) $$\dot{\rho }=-\lambda \left(\rho -\rho_{\infty }\right)-k_{r}\min\left(0,T\left(\frac{e}{\rho }\right)e^{T}u_{c}-∥u-u_{d}∥\right),\;k_{r}>0,$$

where $\lambda ,\rho_{\infty }$ denote the parameters that encapsulate the transient and steady-state output performance specifications.

**Remark** **3.** *Note that the first term in (13) is negative, enforcing the output constraints on the tracking error. However, when the reference tracking task potentially leads to collisions, indicating that the vectors e and $u_{c}$ do not point in the same direction (i.e., $e^{T}u_{c}<0$) or when the control signal $u_{d}$ becomes saturated, the second term in (13) relaxes the performance function ρ(t). This relaxation allows the tracking error to increase, thereby avoiding a collision or mitigating the effects of saturation until all constraints become compatible. Once this compatibility is achieved, the second term is nullified, and the adaptive performance function reverts to its prescribed form, ensuring an exponential convergence rate dictated by the parameter λ.*

### 4.3. Stability Analysis

The main results of the proposed controller are summarized in the following theorem.

**Theorem** **1.** *Consider a unicycle robot operating in a 2D space, governed by the non-affine model (1), while adhering to the input and output performance constraints and navigating among obstacles. Consider also a smooth reference trajectory $p_{d}\left(t\right)$ and that the robot initializes under appropriate conditions ensuring that all constraints are initially satisfied. The proposed control scheme (9)–(13) guarantees the tracking of $p_{d}\left(t\right)$ with adaptive performance specifications, ensuring obstacle avoidance and the boundedness of all closed-loop signals for t≥0.*

**Proof.**  First, let us define the transformed tracking error $\epsilon =T(\frac{e}{\rho })$ and consider the Lyapunov function candidate $V=\frac{1}{2}\epsilon^{2}$. Differentiating with respect to time and substituting (13), we obtain:

(14) $$\dot{V}=D\epsilon \left(e^{T}\dot{e}+\frac{e}{\rho }\lambda \left(\rho -\rho_{\infty }\right)+k_{r}\frac{e}{\rho }\min\left(0,\epsilon e^{T}u_{c}-∥u-u_{d}∥\right)\right)$$

with $D\frac{J(\epsilon )}{\rho (t)}>0$, where J(·) denotes the Jacobian of mapping T(·) and acts as a positive scaling factor.

Note that the term $e^{T}\dot{e}$ is bounded owing to input constraints (2) while $\frac{e}{\rho }\lambda \left(\rho -\rho_{\infty }\right)$ is bounded by construction, leading to:

(15) $$\begin{array}{c} \dot{V}\leq D\epsilon \left(\underset{t\geq 0}{\sup}\left(|e^{T}\dot{e}+\frac{e}{\rho }\lambda \left(\rho -\rho_{\infty }\right)|\right)+k_{r}\frac{e}{\rho }\min\left(0,\epsilon e^{T}u_{c}-∥u-u_{d}∥\right)\right). \end{array}$$

Furthermore, when $e^{T}u_{c}>0$, then the trajectory tracking and collision avoidance goals are compatible. Moreover, noticing (9), it can be easily shown that the term $∥u-u_{d}∥=∥\sigma \left(u_{d}\right)-G^{-1}\left(\theta \right)u_{r}∥$ is a strictly increasing and radially unbounded function of ϵ. Thus, $\dot{V}$ is rendered negative for large ϵ, guaranteeing the ultimate boundedness of the transformed tracking error ϵ.

Additionally, system (1) obeys the input-to-state stability property; hence, it can be shown that ρ(t) remains bounded according to Theorem 1 in [34]. Subsequently, by invoking $T^{-1}\left(\epsilon \right)$, we conclude that e(t)<ρ(t) for all t≥0, ensuring adaptive performance tracking. Consequently, the boundedness of all closed-loop signals implies that $∥p_{r}-p_{o}∥\geq R+r_{o}$, ensuring collision avoidance for all t≥0 and completing the proof. □

## 5. Results

### 5.1. Simulation Results

In this simulation study, we implemented the proposed control scheme (9)–(13), along with the robust velocity estimator (3) and (4), on a nonholonomic robot governed by (1). The robot operates in a planar workspace cluttered with circular obstacles. The simulation is conducted in MATLAB using the ode15s solver, with absolute and relative tolerances set to $10^{-9}$. The parameters for this simulation study are detailed in Table 1.

**Figure 3:**
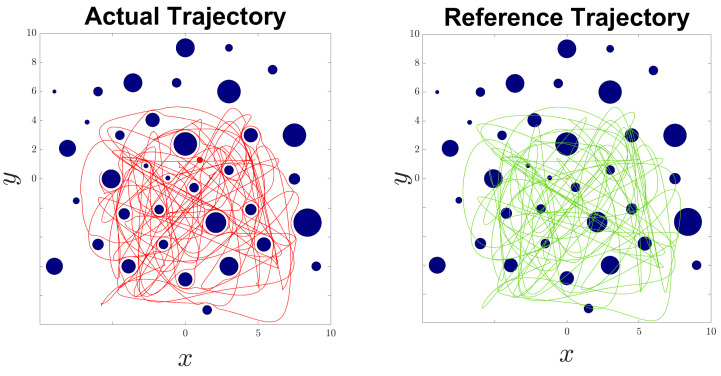
Robot motion: actual trajectory (red line); reference trajectory (green line).

The reference trajectory $p_{d}\left(t\right)$ was generated using the MATLAB cscvn function, which constructs a cubic spline interpolating a sequence of points within the 2D workspace. As shown in the right subfigure of Figure 3, the reference trajectory may pass through obstacles. However, the left subfigure in Figure 3 demonstrates that the actual trajectory of the robot, controlled by the proposed scheme, remains collision-free while closely following the reference path. This illustrates the effectiveness of the control scheme in ensuring safe navigation by dynamically adjusting to avoid obstacles while maintaining adherence to the desired trajectory as closely as possible. The robot’s ability to avoid collisions and track the reference path underscores the robustness and adaptability of the proposed control and estimation algorithm.

**Figure 4:**
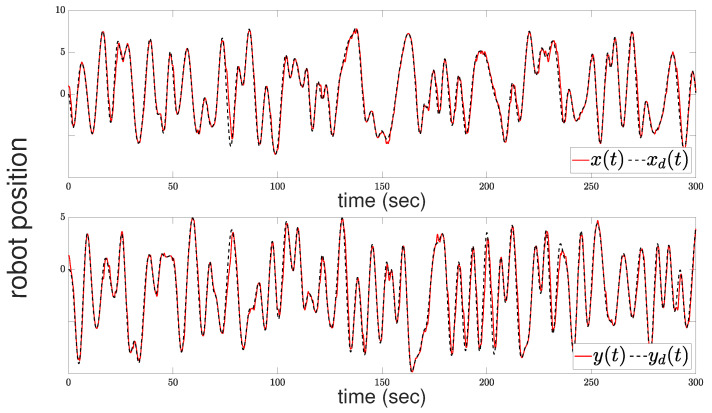
Robot motion: position evolution with respect to axis x (**upper**); position evolution with respect to axis y (**bottom**).

**Figure 5:**
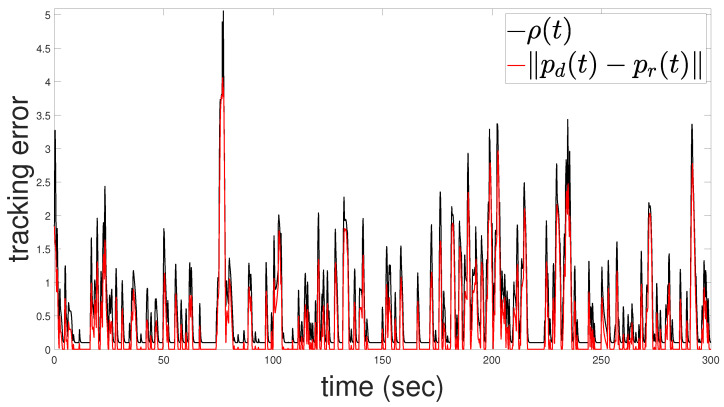
Evolution of tracking error e(t) (red line) vs. the performance function ρ(t) (black line).

Figure 4 illustrates the tracking performance of the robot’s center position with respect to the *x* and *y* axes. The upper subfigure shows the *x*-axis performance, while the lower subfigure displays the *y*-axis performance. In Figure 5, the evolution of the tracking error e(t) is depicted alongside the adaptive performance function ρ(t) governed by (13). The adaptive performance function ρ(t) plays a crucial role in regulating the output performance characteristics. It dynamically adjusts by widening whenever the robot faces the risk of collision and/or when the control input reaches the saturation limits. This relaxation mechanism ensures that the robot avoids collisions and guarantees the boundedness of the closed-loop signals.

Notably, when all constraints are compatible, ρ(t) rapidly returns to its prescribed form, ensuring that the prescribed performance specifications are retained. This adaptability allows the control scheme to balance between maintaining precise tracking and ensuring safety, highlighting the robustness and efficacy of the proposed approach in cluttered environments.

**Figure 6:**
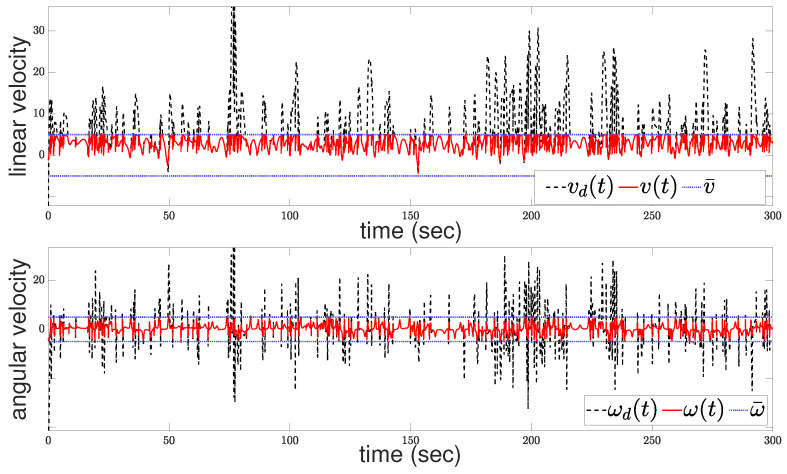
Control commands: linear velocity (**upper**); angular velocity (**bottom**).

Furthermore, Figure 6 presents both the actual, i.e., constrained, and the desired, i.e., unconstrained, control velocities. The upper subfigure illustrates the linear velocities, while the lower subfigure displays the angular velocities. One can observe the intensity of the control effort required due to the presence of multiple constraints, including collision avoidance and input saturation limits. The constrained velocities reflect the adjustments made by the control scheme to ensure these constraints are met. Despite the aggressive nature of the control actions necessitated by the complexity of the problem, the system effectively balances the need for satisfactory tracking with the imperative of maintaining safety and signal boundedness. The discrepancies between the desired and actual velocities underscore the performance adjustments the controller must make to navigate the operational space effectively.

**Figure 7:**
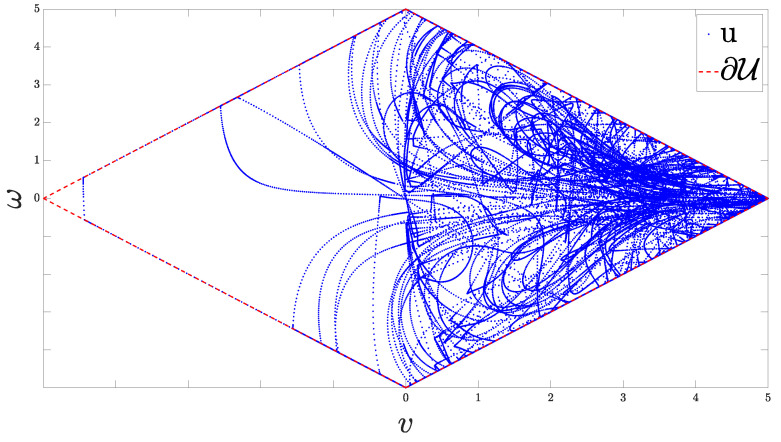
The constrained control input *u* inside the diamond-shaped compact set U.

Figure 7 illustrates the actual control signal *u* plotted within the diamond-shaped compact set U. This figure clearly demonstrates that the control inputs consistently remain within the feasible region defined by U. The diamond-shaped boundary represents the input saturation constraints, which encapsulate the maximum allowable linear and angular velocities, which cannot be applied simultaneously. The control scheme ensures that the applied control effort respects these constraints at all times, effectively balancing the need for the output prescribed performance with the practical limitations of the robot’s actuators. This adherence to the feasible control set U is crucial for the safe and reliable operation of the nonholonomic robot, preventing excessive control actions that could lead to instability or hardware damage.

**Figure 8:**
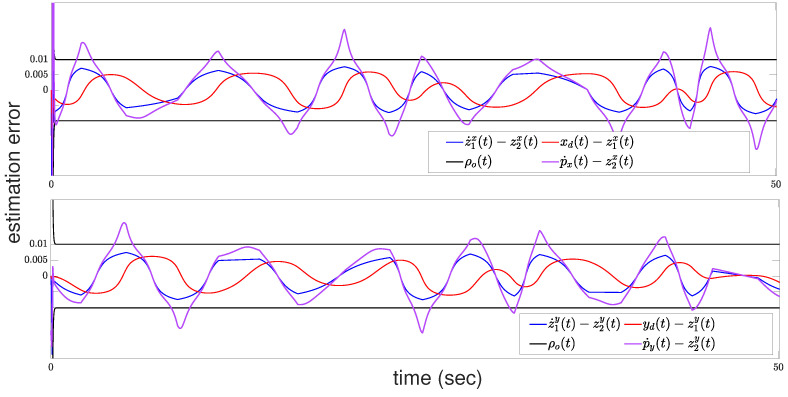
Velocity estimator: estimation errors regarding the velocities with respect to axis x (**upper**) and axis y (**bottom**).

Finally, the performance of the proposed velocity estimator (3) and (4) is depicted in Figure 8, with the velocity estimation errors with respect to axis x,y shown in the upper and subfigures, respectively. To be clearer, Figure 8 depicts the first 50 s of the simulation. For the rest of the simulation time, the estimator exhibits similar performance, and thus, its illustration was omitted. The velocity estimation errors demonstrate the accuracy of the velocity estimator in reconstructing the reference velocities ${\dot{p}}_{d}\left(t\right)$ based on the available position measurements. Furthermore, Figure 8 illustrates the corresponding estimation errors for each axis, which converge to a small neighborhood around zero over time and implicitly determine the performance of the velocity estimation errors ${\dot{p}}_{x}\left(t\right)-z_{2}^{x}\left(t\right)$ and ${\dot{p}}_{y}\left(t\right)-z_{2}^{y}\left(t\right)$. The evolution of the estimation errors are directly dictated by the parameters $\lambda_{o}$ and $\rho_{o}^{\infty }$. The parameter $\lambda_{o}$ dictates the convergence rate, while $\rho_{o}^{\infty }$ determines the steady-state accuracy, representing the error bound in the estimation process. Together, these parameters implicitly regulate the overall estimation performance, underscoring the effectiveness of the proposed velocity estimator in providing reliable and accurate velocity estimates, without extensive tuning procedures.

### 5.2. Comparative Results

In this section, we compare the proposed control scheme with the method proposed in [35]. The reference trajectory is a rose curve given by:

$$\begin{array}{cc} x(t) & =3\cos\left(3t\right)\cos\left(t\right)\\ y(t) & =3\cos\left(3t\right)\sin\left(t\right). \end{array}$$

**Figure 9:**
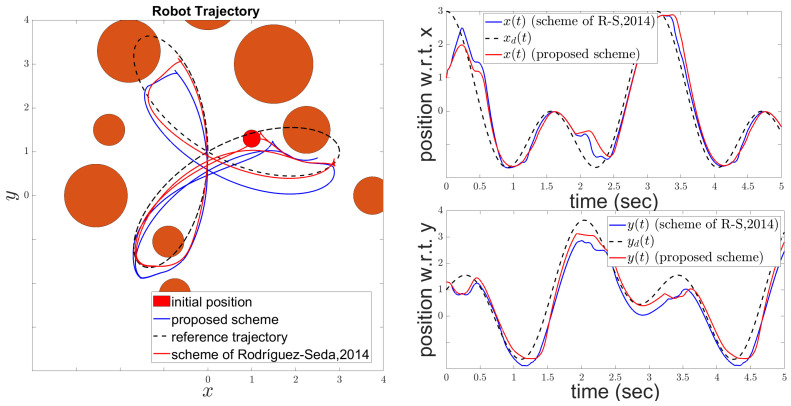
The tracking performance in 2D space (**left**) and with respect to the x- and y-axes (**right**) under the proposed scheme and the method introduced in [35].

**Figure 10:**
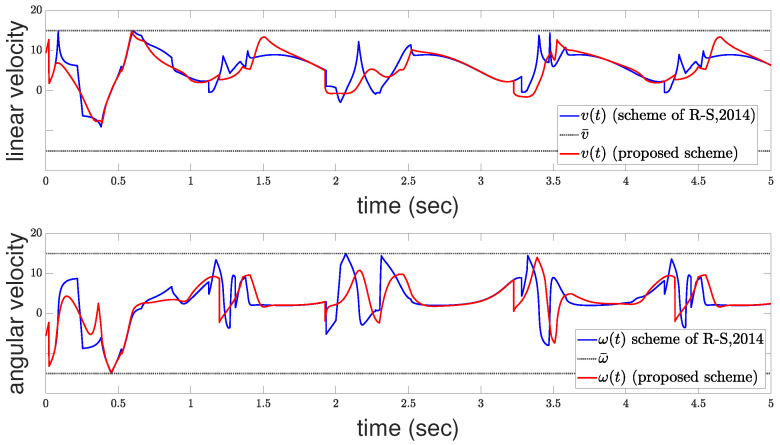
Control commands: linear velocity (**upper**); angular velocity (**bottom**).

The control and performance parameters of the proposed scheme are identical to those listed in Table 1. The control parameters for the method described in [35] were fine-tuned to k=8.23, G=70, and κ=120, with the minimum allowable distance between the robot and obstacles set to $\delta_{r}=0.014$. Additionally, the saturation levels for both schemes were set to $\bar{v}=\bar{\omega }=15$. The resulting tracking performance for both schemes is depicted in Figure 9, while the corresponding control signals are illustrated in Figure 10. Note that the control signals generated by the proposed scheme are less aggressive than those from [35], while also the robot under the proposed controller achieves more accurate trajectory tracking. To further illustrate the superiority of the proposed controller, we provide a performance comparison using three indices that provide distinct insights into the system behavior. The Average Squared Error index (ASE) highlights faster convergence with lower values, emphasizing larger errors. Conversely, the Average Absolute Error index (AAE) indicates slower convergence, but with reduced oscillations. Lastly, the Total Energy Consumption index (TEC) quantifies energy efficiency, where lower values signify more efficient energy utilization during control operations. For a comprehensive analysis of the mathematical formulations underlying the performance indices, please refer to [36].

Note that our approach demonstrates superior tracking performance over the one proposed in [35] as verified by the indices detailed in Table 2.

### 5.3. Experimental Results

**Figure 11:**
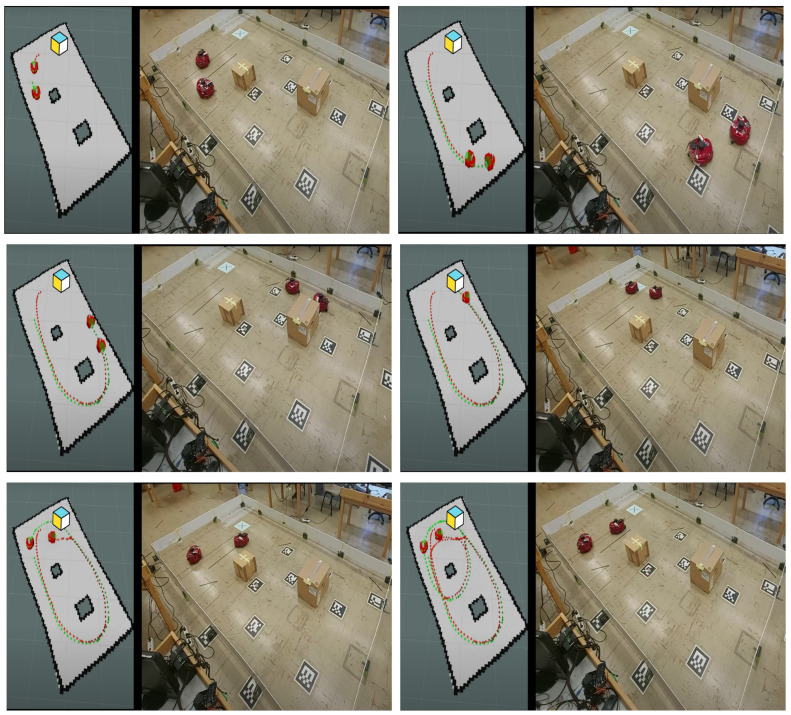
Real-world experiment workspace along with robots and the rviz animation for different time instances.

In this case study, an experiment was conducted in our lab to demonstrate the performance and robustness of the proposed control scheme in a real-world environment. This setting introduces various uncertainties, such as traction variability, measurement noise, and delays inherent in the actuating hardware when commands are issued to the robot. For this experiment, two AmigoBots were utilized, as shown in Figure 11. One robot served as the target, generating the reference trajectory, while the other robot was forced to track this trajectory. The entire experiment was implemented using the ROS framework and Python to control the robots. Both robots were equipped with LiDAR sensors and Odroid units (mini computers) running Ubuntu. The target robot moved within the workspace via tele-operation, simulating dynamic trajectory changes that the tracking robot had to follow.

The LiDARs were utilized for SLAM, for both robots, measuring their position in order to obtain the tracking error e(t). Subsequently, the second robot was equipped with the proposed tracking controller, with the following parameters. The performance function parameters were set as λ=0.5 and $\rho_{\infty }=0.1$. The rest of the parameters were chosen as $k_{d}=0.15,\;k_{o}=1,\;k_{r}=0.5,\;r=0.3,\;a_{1}=1,\;a_{2}=1,\;\lambda_{o}=30,\;\rho_{o}^{\infty }=0.01$. Finally, the saturation limits were $\bar{v}=0.3\;m/s$ and $\bar{\omega }=0.8\;\mathrm{rad}/s$. Moreover, in order to showcase the efficacy of the proposed control scheme to ensure collision avoidance even in case the target crosses within an obstacle, we considered a virtual obstacle marked with the light blue square in Figure 11. The target could cross the virtual obstacle, but the robot had to properly steer to avoid this danger zone.

**Figure 12:**
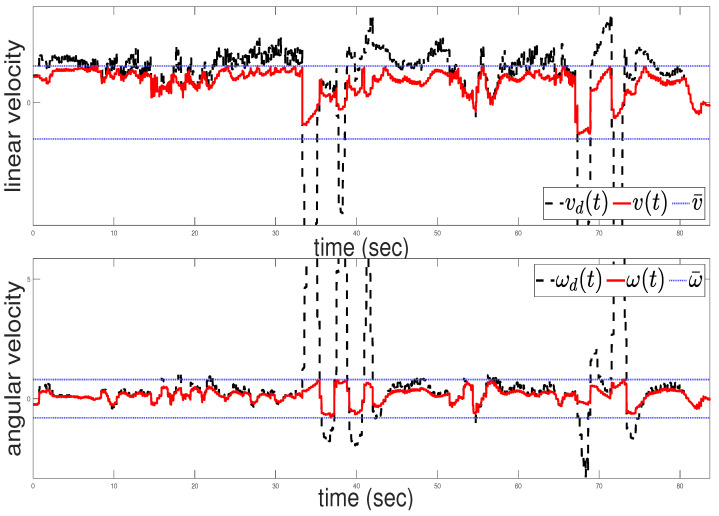
Control commands: linear velocity (**upper**); angular velocity (**bottom**).

A video showcasing the real-world experiment and the tracking performance can be accessed via the following link: https://www.youtube.com/watch?v=Sty73Vd3SMQ (accessed on 2 June 2024). Figure 12 depicts the commanded velocity of the robot throughout the experiment. Notably, the upper subfigure in Figure 12 shows instances where the commanded linear velocity becomes abruptly negative at approximately the 35-th and 68-th seconds, resulting in backward motion. This behavior, observable in the linked video, is attributed to the presence of the virtual obstacle that significantly increases the collision avoidance term $u_{c}$, preventing the robot from approaching the obstacle. This highlights the importance of selecting smaller control gains $k_{o}$ to enhance the system’s response in such scenarios. It can be concluded that, in the real-world scenario, the commanded velocities given by the control scheme are more oscillatory than those in the simulation study. This phenomenon can be attributed to practical constraints such as slip, as well as delays introduced by measurements, both of which hinder the algorithm’s performance, as expected.

## 6. Conclusions

In this study, we addressed the trajectory-/target-tracking problem for a unicycle robot operating within a cluttered 2D space with obstacles. The proposed control scheme integrates multiple constraints, including input–output and safety constraints, into a unified performance function that governs the overall motion of the robot. Given the critical significance of both safety and the input constraints, the control scheme dynamically adjusts the output performance constraints whenever conflicts arise with safety and/or the input constraints. This adaptive mechanism guarantees the robot’s effective and safe operation across diverse operational conditions. Additionally, we introduced a novel velocity estimator to accurately reconstruct the unmeasured velocity of the reference trajectory/target. This estimator enhances the tracking precision of the robot by providing reliable velocity estimates, thereby enabling it to effectively track dynamic reference trajectories or targets even in the absence of direct velocity measurements.

In the future, our research endeavors will concentrate on considering feedback delays and incorporating state constraints to improve the efficiency and practicality of the proposed control protocol. Additionally, we aim to delve deeper into scenarios involving target loss resulting from environmental obstructions, such as motion blur or varying light conditions. Furthermore, there is a need for further investigation into automating the tuning of all control gains while also considering the dynamics of the robot.

## Figures and Tables

**Table 1:** Simulation parameters.

Parameter	Value	Parameter	Value	Parameter	Value
*R*	0.2	$k_{d}$	3	ρ(0)	1.84
*r*	0.3	$k_{o}$	1	$a_{i},\;i=1,2$	1
$\bar{v}$	5	$k_{r}$	0.5	$\lambda_{o}$	30
$\bar{\omega }$	5	λ	5	$\rho_{o}^{\infty }$	0.01
δ	0.016	$\rho_{\infty }$	0.1	$\rho_{o_{i}}\left(0\right),\;i=1,2$	1

**Table 2:** Tracking performance indices.

Performance Index	Proposed Scheme	Scheme of [35]
$\mu_{\mathrm{ASE}}$	0.67052	0.75987
$\mu_{\mathrm{AAE}}$	0.88837	0.94335
$\mu_{\mathrm{TEC}}$	11.4347	12.157

## Data Availability

The original contributions presented in the study are included in the article, further inquiries can be directed to the corresponding author.

## Abbreviations

The following abbreviations are used in this manuscript:

APC	adaptive performance control
APF	artificial potential field
EKF	Extended Kalman Filter
EMPC	event-triggered model predictive control
LiDAR	Light Detection And Ranging
PF	performance function
PI	proportional–integral
PID	proportional–integral–derivative
PPC	Prescribed Performance Control
ROS	Robot Operating System
FLC	fuzzy logic controller
MPC	model predictive control
NMPC	nonlinear model predictive control
BLF	Barrier Lyapunov Function
WMR	wheeled mobile robot
